# Relationships between Serum Uric Acid, Malondialdehyde Levels, and Carotid Intima-Media Thickness in the Patients with Metabolic Syndrome

**DOI:** 10.1155/2019/6859757

**Published:** 2019-10-09

**Authors:** Shun-Sheng Wu, Chew-Teng Kor, Ting-Yu Chen, Ko-Hung Liu, Kai-Lun Shih, Wei-Wen Su, Hung-Ming Wu

**Affiliations:** ^1^Department of Gastroenterology, Changhua Christian Hospital, Changhua, Taiwan; ^2^Internal Medicine Research Center, Changhua Christian Hospital, Changhua, Taiwan; ^3^Inflammation Research & Drug Development Center, Changhua Christian Hospital, Changhua, Taiwan; ^4^Department of Neurology, Changhua Christian Hospital, Changhua, Taiwan; ^5^Graduate Institute of Acupuncture Science, China Medical University, Taichung, Taiwan

## Abstract

Oxidative stress is the major cause of atherosclerosis and cardiovascular diseases. This cross-sectional study is aimed at determining if parallel serum markers of oxidative stress are related to carotid intima-media thickness (IMT). We enrolled 134 participants with varied metabolic syndrome (Met-S) scores (zero, *n* = 21; one, *n* = 19; two, *n* = 27; three, *n* = 26; four, *n* = 25; five, *n* = 16). Biochemical profiles and potential oxidative stress biomarkers malondialdehyde (MDA) and uric acid were measured in fasting plasma. We found that carotid IMT positively correlated with both MDA and uric acid levels. Multivariate analysis revealed that both MDA (*p* < 0.05) and uric acid (*p* < 0.01) levels were significantly associated with carotid IMT in participants whose Met-S scores were ≥1 or ≥2. However, only uric acid (*p* < 0.01) levels were positively associated with carotid IMT in patients with metabolic syndrome. Linear regression model analysis revealed that the prediction accuracies for carotid IMT from MDA combined with uric acid and from a combination of MDA, uric acid, and Met-S score were 0.176 and 0.237, respectively. These were better than the predication accuracies from MDA (*r*^2^ = 0.075) and uric acid (*r*^2^ = 0.148) individually. These results suggest that measuring uric acid levels along with MDA biomarkers and Met-S scores may be a promising step in the development of an effective model for monitoring the severity of carotid IMT and atherosclerosis in the patients with metabolic syndrome.

## 1. Introduction

Oxidative stress plays a crucial role in the pathophysiological processes of several diseases, including atherosclerosis [1]. Metabolic syndrome is a cluster of conditions that includes abdominal obesity, high blood pressure, high blood sugar, high serum triglycerides, and low high-density lipoprotein cholesterol (HDL-C) levels. Accordingly, each of these conditions including obesity, dyslipidemia, hypertension, and hyperglycemia carries an independent risk for cardiovascular disease and atherosclerosis [2–5]. An increasing number of studies confirm that metabolic dysregulation causes increases in oxidative stress, contributing to the pathogenesis of atherosclerotic changes [6, 7].

Serum uric acid and malondialdehyde (MDA) are two important biomarkers of oxidative stress [8–10]. In particular, MDA is the most frequently used indicator of oxidative damage to cells and tissue in several conditions (e.g., diabetes). Several studies have shown that elevated serum uric acid levels are associated with conditions of metabolic dysregulation such as hyperlipidemia [11], hypertension, and cardiovascular risk-related factors [12, 13]. MDA is a toxic product of aldehydes from lipid peroxidation. High serum concentration of MDA is associated with metabolic dysregulation of glucose and lipid profiles [6].

Carotid intima-media thickness (IMT), measured noninvasively by ultrasonography, is currently a widely used marker for atherosclerotic disease. Carotid IMT is directly associated with an increased risk of cardiovascular disease [14, 15]. Although previous studies have reported that high levels of MDA and uric acid are individually associated with increased carotid IMT in patients with hypertension and metabolic syndrome [16–18], whether uric acid, or MDA, or their combination may serve as a better predictor of carotid IMT among the patients with metabolic syndrome has not been clarified yet. Therefore, this study was designed to address this issue. Our data show that the prediction accuracies for carotid IMT from MDA combined with uric acid in the cases with higher metabolic syndrome scores were better than those from MDA and uric acid individually.

## 2. Participants and Methods

### 2.1. Study Design and Participants

This study was designed as a cross-sectional case-control study, conducted at the Health Management Center at the Changhua Christian Hospital over a two-year period. All participants aged between 45 and 60 years were eligible for inclusion. Exclusion criteria included evidence of hyperthyroidism, hypothyroidism, alcoholism, or viral hepatitis (type B or C). Cases were also excluded if the patients were receiving antidiabetic medication, statins, antioxidants, vitamin C, vitamin E, nonsteroidal anti-inflammatory drugs, or hepatotoxic agents during the study period. Waist circumference and blood pressure were measured in all participants. After an >8-hour overnight fast, venous blood specimens were obtained from all subjects for biochemical profiles as well as high-sensitivity C-reactive protein (hs-CRP), uric acid, and MDA levels; the information of anthropometric measurements and metabolic syndrome-associated profiles were also obtained. The study was conducted in strict accordance with guidelines for research involving human subjects developed by the Taiwan Ministry of Health and Welfare. All study protocols were approved by the Institutional Review Board of the Changhua Christian Hospital (Approval Number 110507). All participants provided written informed consent to participate in the study.

### 2.2. Definition of Metabolic Syndrome (Met-S) Scores

In this study, metabolic syndrome was defined according to the modified National Cholesterol Education Program (NCEP) criteria with Asian-specific cutoffs [19]. Briefly, metabolic syndrome was diagnosed in patients with ≥ three of the following five components: (1) waist circumference (≥90 cm for men and ≥80 cm for women), (2) blood pressure (systolic ≥ 130 mmHg and/or diastolic ≥ 85 mmHg), (3) triglycerides (≥150 mg/dl), (4) HDL-C (<40 mg/dl in male and <50 mg/dl in female), or (5) fasting glucose (≥100 mg/dl). The participants were initially stratified into Met-S score subgroups according to the number of Met-S components.

### 2.3. Anthropometric Measures

Blood specimens were obtained from participants in the morning following an overnight fast. The plasma was aliquoted and stored at −80°C, without thawing until assay. A history of cigarette and alcohol use was obtained from each study participant. Height and weight were measured in light clothing without shoes. BMI was calculated as weight (kg)/height^2^ (m)^2^.

### 2.4. Carotid Ultrasonography to Measure Intima-Media Thickness of the Common Carotid Arteries

IMT of the common carotid arteries was assessed using high-resolution B-mode ultrasonography (Acuson 128XP, equipped with a 7 MHz linear array transducer). All measurements were conducted by the same experienced sonographer on a day close to the day of blood biochemistry analysis. The IMT value was defined as the mean of 10 IMT measurements on the far wall of the bilateral common carotid arteries about 10 mm proximal to the carotid bifurcation. The lumen/intima leading edge (I-line) to media/adventitia leading edge (M-line) method was used, which is validated anatomically as previously described [20].

### 2.5. Assays for Plasma MDA and Plasma Superoxide Dismutase

Plasma MDA was assayed with a thiobarbituric acid reactive substances assay kit (Cayman Chemical Company, Ann Arbor, MI, USA) according to the manufacturer's instructions. Absorbance of the samples was measured at 532 nm by a microplate reader (Versa Max, Molecular Devices, Sunnyvale, CA, USA). MDA level was determined by using an MDA standard curve. The superoxide dismutase (SOD) is an enzyme protecting lipid from superoxide-induced oxidative stress. The activity (U/ml) of plasma SOD was therefore quantified in order to characterize the antioxidant capability of a biological system using a superoxide dismutase assay kit (Cayman Chemical Company, MI, USA) according to the manufacturer's instructions. The interassay and intra-assay laboratory coefficients of variation for MDA were 5.8% and 8.0%, respectively, and for SOD were 7.2% and 6.5%, respectively.

### 2.6. Assays for Oxidative DNA Damage

Increased oxidative stress may lead to oxidative DNA damage, which may in turn bring about cell injury and death. 8-hydroxy-2′-deoxyguanosine (8-OHdG) is a biomarker that is widely used to indicate the oxidative stress-induced single nucleotide-based lesions [21]. *γ*-H2AX is a protein involved in the first step for repairing DNA double-strand breaks and is a sensitive marker of DNA damage and repair [22]. Quantitation of those two proteins (8-OHdG and *γ*-H2AX) was performed by 8-OHdG assay kit (Wuhan Fine Biotech Co. Hubei, China) and *γ*-H2AX assay kit (Wuhan Fine Biotech Co. Hubei, China), according to the manufacturer's instructions, respectively. The interassay and intra-assay laboratory coefficients of variation for 8-OHdG were 6.8% and 7.5%, respectively, and for *γ*-H2AX were 8.5% and 9%, respectively.

### 2.7. Biochemistry Assays and Other Measures

Serum concentrations of aspartate transaminase (AST), alanine transaminase (ALT), uric acid, fasting blood sugar, hs-CRP, total white blood cell count (WBC), and lipid profiles including total cholesterol, triglyceride, low-density lipoprotein cholesterol (LDL-C), and HDL-C were measured using standard procedures at the Department of Laboratory Medicine, Changhua Christian Hospital. In addition, glycated hemoglobin (HbA1c) levels in venous bloods samples were analyzed by the D-100 HbA1c test (Bio-Rad Laboratories, Inc., CA). The interassay and intra-assay laboratory coefficients of variation for HbA1c varied between 1.1 and 2.3%.

### 2.8. Statistical Analysis

**Figure 1:**
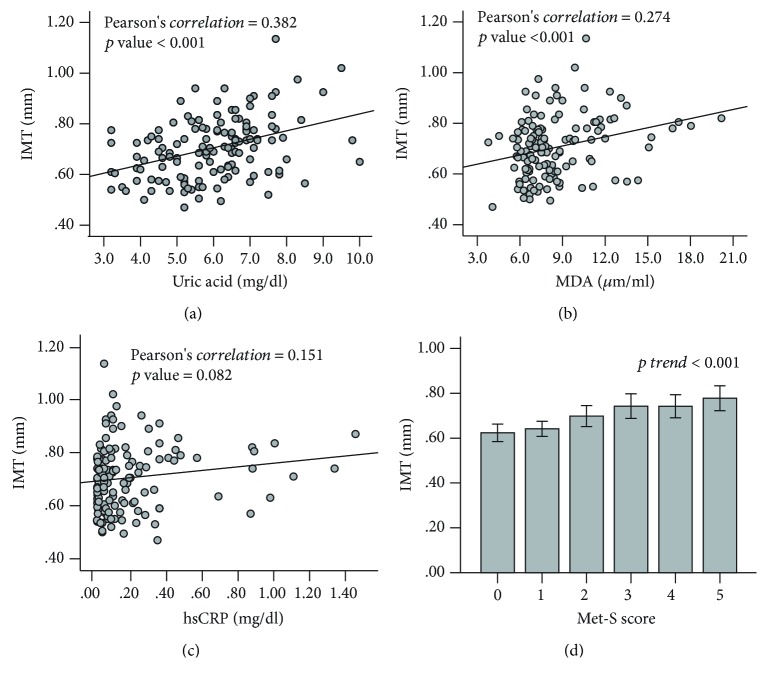
Correlations between carotid IMT and malondialdehyde, uric acid, hs-CRP, and metabolic syndrome scores in all 134 participants. Associations of carotid IMT are positive with uric acid (a), malondialdehyde (MDA) (b), and high-sensitivity C-reactive protein (hs-CRP) (c) using Pearson's correlation test, and with metabolic syndrome (Met-S) scores (d) using one-way ANOVA analysis.

**Figure 2:**
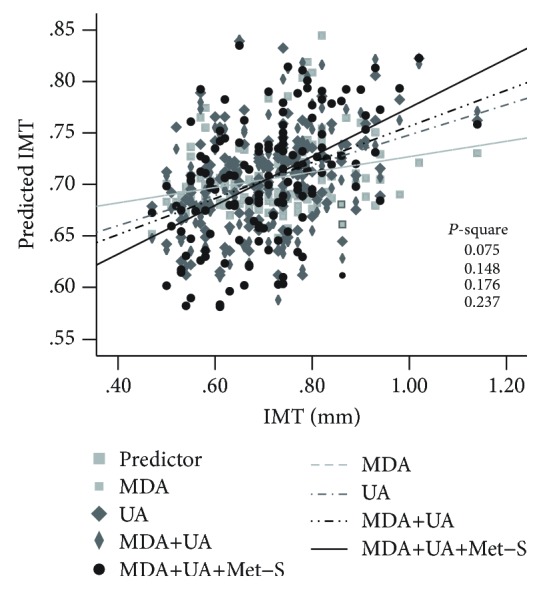
The prediction accuracy (*r*^2^) of carotid IMT variance for malondialdehyde (MDA), uric acid, and metabolic syndrome (Met-S) scores in all 134 participants by using a linear regression model.

Data are represented as mean ± standard deviation (SD). The chi-square test was used for categorical comparisons of data, and the ANOVA test was used to measure differences in means of continuous variables between the six Met-S score subgroups (Table 1 and Figure 1). The Jonckheere-Terpstra test was used to test for an ordered alternative hypothesis within six subgroups (Table 1). The Pearson correlation analysis was performed to evaluate the correlation between carotid IMT and oxidative stress biomarkers, as well as Met-S scores (Figure 1). The multiple linear regression model was used to assess the relationships between oxidative stress biomarkers (Tables 2 and 3), and conditions of metabolic dysregulation, as well as carotid IMT (Table 4). A linear regression model was used to calculate the predicted values of carotid IMT (Figure 2). A *p* value < 0.05 was considered as an indicator of significant statistical difference. All statistical analyses were conducted using the statistical package SPSS (IBM SPSS Statistics, version 20, IBM Corporation, Chicago, IL, USA).

## 3. Results

### 3.1. Demographic, Clinical, and Laboratory Data

This study enrolled 134 participants who visited the Health Management Center at the Changhua Christian Hospital for health management reasons over a two-year period. Table 1 shows demographic, clinical, and laboratory data from participants in the six Met-S score subgroups. There were no significant differences in age, gender, alcohol use, or cigarette use among these subgroups. WBC, hs-CRP, average IMT, GOT, and GPT showed graded increasing trends, while HDL-C showed a graded decrease (Table 1).

### 3.2. Increased Levels of Oxidative Stress Markers and Lower Activity of Antioxidant Enzymes in the Subgroups with Higher Met-S Scores

The plasma levels of uric acid and lipid peroxidation product MDA were significantly increased in parallel with the number of Met-S scores (Table 1). The activity of antioxidant enzyme SOD showed the tendency toward decrease among Met-S subgroups, whereas both 8-OHdG and *γ*-H2AX levels of oxidative DNA damage showed the tendency toward increase (Table 1). The plasma level of MDA was significantly correlated with 8-OHdG levels (*r* = 0.2418, *p* = 0.0089).

### 3.3. Correlations between Carotid IMT, Uric Acid, MDA, hs-CRP, and Metabolic Syndrome Scores

We examined the relationships between carotid IMT and the oxidative stress biomarkers, MDA and uric acid, and the inflammatory factor, hs-CRP, in all 134 participants using Pearson's correlation test. The analysis revealed that carotid IMT positively correlated with uric acid (*r* = 0.382, *p* < 0.001) and MDA (*r* = 0.274, *p* < 0.001), but not significantly correlated with hs-CRP (*r* = 0.151, *p* = 0.082) (Figures 1(a)–1(c)). We also found that carotid IMT showed significant correlation with Met-S scores (*r* = 0.225, *p* trend < 0.001) (Figure 1(d)).

### 3.4. Relationship of Oxidative Stress Biomarkers (MDA and Uric Acid) with Metabolic Components

Reports have suggested that oxidative stress is associated with conditions of metabolic syndrome and several traditional risk factors [6, 7]. Therefore, we examined which factors are closely associated with MDA and uric acid. After adjusting for the five conditions of metabolic syndrome and traditional risk factors, multivariate analysis revealed that MDA was significantly correlated with fasting blood sugar (*p* < 0.001), Met-S score (*p* < 0.001), and triglycerides (*p* = 0.005) as well as negatively with HDL-C (*p* = 0.014) (Table 2). Uric acid was positively correlated with diastolic blood pressure (*p* = 0.001) and metabolic score (*p* < 0.001) as well as negatively with HDL-C (*p* = 0.028) (Table 3).

### 3.5. Multivariate Analysis to Evaluate the Associations among MDA, Uric Acid, and Metabolic Syndrome Traits and Carotid IMT

We then examined the role of these two oxidative stress biomarkers in association with carotid IMT in our study participants. Multivariate analysis revealed that Met-S score (*p* = 0.044) and uric acid (*p* = 0.013) were positively associated with carotid IMT among all participants (*n* = 134), after adjusting for traditional risk factors (age, gender, smoking, and alcohol use). However, MDA and hs-CRP were not positively associated with carotid IMT (Table 4). Stratified by Met-S scores, further analysis revealed that both MDA (*p* < 0.05) and uric acid (*p* < 0.01) levels were significantly associated with carotid IMT in participants whose Met-S scores were ≥1 or ≥2. However, only uric acid (*p* < 0.01) was positively associated with carotid IMT in participants with metabolic syndrome, i.e., patients with Met-S scores ≥ 3.

### 3.6. Uric Acid Combined with MDA and Met-S Score Is Better Predictive of Severity of Carotid IMT

Finally, the linear regression model was used to calculate the predicted values of carotid IMT based on the total variation of carotid IMT. We observed that the prediction accuracy (*r*^2^) of carotid IMT variance for MDA and uric acid was 0.075 and 0.148, respectively. The prediction accuracy (*r*^2^) of carotid IMT variance for MDA combined with uric acid and for a combination of MDA, uric acid, and Met-S score was 0.176 and 0.237, respectively (Figure 2).

## 4. Discussion

In this study, we evaluated the association between oxidative stress biomarkers and carotid IMT, and whether combination of these biomarkers is a better predictor for carotid IMT in our study participants. We found that participants with higher metabolic syndrome scores, MDA, and uric acid levels had significantly higher levels of carotid IMT (all *p* values < 0.001 and *p* trend < 0.001). Multiple regression analysis showed that both MDA and uric acid were significantly associated with carotid IMT in participants with ≤2 Met-S components. Furthermore, in participants with metabolic syndrome, uric acid was an independent factor of carotid IMT. An evaluation of both oxidative stress biomarkers (MDA plus uric acid) or evaluating them in combination with Met-S scores might provide more accurate measurements of carotid IMT.

The present study showed consistent results in the increased levels of oxidative stress products and its related DNA damage markers (e.g., 8-OHdG and *γ*-H2AX), and in the lower activity of antioxidant enzyme (e.g., SOD), supporting the increase of oxidative stress in metabolic syndrome and its component pathologies [6, 7, 12, 22, 23]. However, the mechanism underlying oxidative stress in metabolic syndrome remains unclear. We revealed the significant correlation of total WBC counts with serum MDA (*r* = 0.3511, *p* < 0.0001, Pearson's test) and uric acid (*r* = 0.1944, *p* = 0.0244, Pearson's test), respectively. Given evidence indicates that phagocytic NADPH oxidases in leukocytes become overactive in the metabolic syndrome patients, which has been considered as the primary source of reactive oxygen species (ROS) involved in atherosclerosis [7, 24]. Since phagocytic NADPH oxidases are predominantly expressed in the innate immune cells [25], total WBC count might serve as a nontraditional potential biomarker for lipid peroxidation and atherosclerosis in the patients with metabolic disorders [25–27].

Uric acid is the end product of purine metabolism in human beings and higher primates. Although it has been suggested that uric acid can act as an antioxidative scavenger, providing powerful free radical scavenging capacity in plasma [28, 29], it may also act as a prooxidant to trigger oxidative stress in cells and contribute to endothelial dysfunction and damage via triggering oxidative and endoplasmic reticulum stress and inducing mitochondrial dysfunction and mitochondrial DNA damage [30, 31]. Despite the reported beneficial role of uric acid [28, 29], this study suggests that higher levels of uric acid are correlated with carotid IMT in participants with various conditions of metabolic dysregulation (Table 4) [32].

Due to a molecular structure that is abundant with reactive double bonds, lipids are susceptible targets of oxidation [33, 34]. MDA is one of the main products of lipid peroxidation. It is also a toxic molecule with oxidized low-density LDL. Therefore, it promotes atherosclerosis [35, 36]. Consistent with previous studies [37, 38], our study suggests that elevated levels of MDA are correlated with carotid IMT. We further reveal that this association was closely observed in subjects with fewer metabolic syndrome components (Table 4). This might imply that more complicated mechanisms are involved in advanced stages of atherosclerosis, although higher MDA levels tend to be associated with having more conditions within the metabolic syndrome.

A number of studies have shown that MDA and uric acid are individual risk factors for increase of carotid IMT and atherosclerosis [39–41]. However, few studies have been conducted to show how both parallel biomarkers simultaneously contribute to carotid atherosclerosis and its progression [42]. Our study, to the best of our knowledge, is the first to report that the presence of circulating uric acid combined with MDA in participants with varied metabolic syndrome scores significantly increased the risk of carotid IMT (Figure 2). Interestingly, our study showed that MDA and uric acid had different relationship profiles with carotid IMT, metabolic syndrome conditions, and traditional risk factors. Lipid peroxidation MDA level was predominantly associated with lipid disorders (e.g., high LDL-C), high blood sugar levels, and metabolic scores (Table 2). Although glucose is not a lipid, it can be converted from the glycerol component of triglycerides, in particular, in patients with metabolic syndrome and diabetes. In contrast, uric acid was associated with hypertension, TGs, and metabolic scores (Table 3). The comparative relationships of uric acid and MDA to carotid IMT might suggest that a diagnostic model comprised of Met-S score and those two oxidative stress serum biomarkers may provide an effective strategy for detecting and monitoring the development of atherosclerosis.

There are several limitations in this study. Firstly, this study was a cross-sectional study. Therefore, it did not allow for determination of causal relationships. Secondly, the study had a relatively small sample size, which may have reduced its statistical power. Longitudinal studies with a larger sample size are needed to establish cause-effect relationships in the future. Thirdly, the information of alcohol and cigarette use, which are considered as common causes of oxidative stress for cardiovascular disease, was obtained by self-reported questionnaire. It might be underestimated for alcohol and cigarette use in our participants.

## 5. Conclusion

We found that oxidative stress biomarkers, increased levels of MDA and uric acid, were positively associated with increased carotid IMT in cases with multiple metabolic syndrome conditions. Specifically, we found that the combination of Met-S scores with uric acid and MDA was the most accurate measure for predictive IMT values. A diagnostic model comprising of the clinical features, metabolic components, and serum oxidative stress biomarkers may provide an effective strategy for detecting and monitoring the development of atherosclerosis. Longitudinal studies are needed to support our novel findings.

## Figures and Tables

**Table 1:** Demographic, clinical, and laboratory data of the stratified subgroups by metabolic syndrome score.

	Met − S score = 0	Met − S score = 1	Met − S score = 2	Met − S score = 3	Met − S score = 4	Met − S score = 5	*p* value	*p* trend
Sample size, *n*	21	19	27	26	25	16		
Age (year)	49.67 ± 4.85	50.21 ± 4.42	52.93 ± 5.87	50.88 ± 5.74	51.24 ± 6.08	53.19 ± 6.84	0.264	0.089
Male, *n* (%)	7 (33.33%)	10 (52.63%)	17 (62.96%)	12 (46.15%)	13 (52%)	10 (62.5%)	0.383	0.214
Smoking, *n* (%)	1 (4.76%)	1 (5.26%)	3 (11.11%)	3 (11.54%)	4 (16%)	5 (31.25%)	0.189	0.015
Alcohol, *n* (%)	3 (14.29%)	3 (15.79%)	8 (29.63%)	7 (26.92%)	6 (24%)	4 (25%)	0.792	0.344
BMI (kg/m^2^)	22.13 ± 2.78	23.61 ± 1.96	24.84 ± 3.01	25.85 ± 2.7	27.55 ± 3.32	29.44 ± 3.74	<0.001	<0.001
Waist (cm)	74.6 ± 6.28	78.66 ± 6.02	85.39 ± 8.57	84.48 ± 6.57	92.21 ± 8.77	97.88 ± 9.33	<0.001	<0.001
SBP (mmHg)	107.52 ± 8.9	122.74 ± 13.08	125.93 ± 15.35	130.73 ± 17.36	139.04 ± 12.24	145.88 ± 16.3	<0.001	<0.001
DBP (mmHg)	71.14 ± 6.36	78.26 ± 7.85	79.67 ± 8.06	84.92 ± 13.41	88.16 ± 8.54	88.5 ± 7.92	<0.001	<0.001
Uric acid (mg/dl)	5.1 ± 1.4	5.5 ± 1.4	6.05 ± 1.44	6.04 ± 1.07	6.46 ± 1.53	6.78 ± 0.7	0.001	<0.001
MDA (*μ*m/ml)	6.96 ± 1.17	6.98 ± 1.3	7.62 ± 2.11	8.54 ± 2.05	9.09 ± 1.9	13.02 ± 3.78	<0.001	<0.001
SOD (U/ml)	4.06 ± 0.37	3.88 ± 0.31	3.59 ± 0.36	3.26 ± 0.34	2.58 ± 0.32	2.42 ± 0.36	0.0073	<0.001
8-OHdG (ng/ml)	36.97 ± 6.74	55.72 ± 8.36	70.04 ± 8.21	77.46 ± 9.62	81.38 ± 5.01	90.06 ± 6.76	<0.001	<0.001
*γ*-H2AX (pg/ml)	29.97 ± 4.14	34.53 ± 5.09	36.74 ± 6.029	42.52 ± 5.23	53.23 ± 4.82	54.05 ± 4.02	0.0061	<0.001
hs-CRP (mg/dl)	0.05 ± 0.06	0.06 ± 0.08	0.12 ± 0.13	0.22 ± 0.28	0.25 ± 0.32	0.49 ± 0.4	<0.001	<0.001
Fasting blood sugar (mg/dl)	87.1 ± 5.86	93.74 ± 4.39	93.48 ± 8.53	104.15 ± 31.94	111.56 ± 19.93	160.94 ± 57.86	<0.001	<0.001
Hemoglobin A1c (%)	5.19 ± 0.21	5.37 ± 0.4	5.48 ± 0.53	5.81 ± 1.02	6.12 ± 1.04	8.14 ± 2.33	<0.001	<0.001
Total cholesterol (mg/dL)	190.48 ± 31.01	212.74 ± 32.37	205.11 ± 49.95	205.46 ± 35.44	207.44 ± 38.24	229 ± 68.79	0.189	0.035
HDL-C (mg/dL)	56.86 ± 8.74	54.68 ± 14.11	48.22 ± 10.82	45.54 ± 8.31	39.52 ± 7.95	38 ± 8.07	<0.001	<0.001
Triglyceride (mg/dL)	70.05 ± 21.8	95.95 ± 44.18	126.37 ± 63.75	151.81 ± 63.98	215.96 ± 94.92	233.44 ± 109.11	<0.001	<0.001
LDL-C (mg/dL)	114.99 ± 26.63	134.63 ± 27.91	127.12 ± 34.36	129.35 ± 30.38	125.07 ± 45.09	138.81 ± 35.91	0.356	0.160
Tchol_HDLratio	3.39 ± 0.58	4.06 ± 0.93	4.32 ± 0.9	4.63 ± 1.07	8.94 ± 17.52	6.36 ± 2.81	0.148	0.045
WBC (mm^3^)	4.74 ± 1.22	4.83 ± 1.42	5.49 ± 1.32	5.7 ± 1.51	6.81 ± 1.33	7.53 ± 2.56	<0.001	<0.001
AST (U/L)	25.86 ± 9.32	23.47 ± 4.94	27.67 ± 7.68	26.23 ± 7.91	36.36 ± 19.41	34.88 ± 15.2	<0.001	<0.001
ALT (U/L)	21.05 ± 8.84	23.84 ± 7.75	31.26 ± 17.01	29.73 ± 15.53	47.36 ± 28.42	41.19 ± 18.04	<0.001	<0.001
Creatinine (mg/dl)	0.71 ± 0.17	0.76 ± 0.17	0.81 ± 0.16	0.72 ± 0.2	0.78 ± 0.16	0.8 ± 0.18	0.251	0.246
Renal GFR (ml/min/1.73 m^2^)	100.07 ± 22.36	99.15 ± 19.18	95.83 ± 15.75	106.86 ± 29.99	93.77 ± 16.03	97.59 ± 27.49	0.384	0.681
avgIMT (mm)	0.62 ± 0.09	0.64 ± 0.07	0.70 ± 0.12	0.74 ± 0.14	0.74 ± 0.12	0.78 ± 0.1	<0.001	<0.001

Data are presented as mean ± SD or *n* (%) for categorical data. Differences in mean values of variables between the four Met-S-stratified subgroups were tested by one-way ANOVA test. *p* for trend was calculated by the Jonckheere-Terpstra Test to test for an ordered alternative hypothesis within six subgroups. Met-S: metabolic syndrome; BMI: body mass index; SBP: systolic blood pressure; DBP: diastolic blood pressure; WBC: white blood cell; ALT: alanine aminotransferase; AST: aspartate aminotransferase; HDL-C: high-density lipoprotein cholesterol; LDL-C: low-density lipoprotein cholesterol; hs-CRP: high-sensitivity C-reactive protein; MDA: malondialdehyde; avgIMT: average of intima-media thickness of both common carotid arteries; SOD: superoxide dismutase; 8-OHdG: 8-hydroxy-2′-deoxyguanosine.

**Table 2:** Associations of MDA with metabolic components and traditional factors (*n* = 134).

Variables	Model 1		Model 2		Model 3	
	Standardized coefficient beta	*p* value	Standardized coefficient beta	*p* value	Standardized coefficient beta	*p* value
Waist	0.077	0.327				
SBP	0.136	0.193				
DBP	-0.133	0.169				
Fasting blood sugar	0.481	<0.001	0.528	<0.001		
HDL-C	-0.152	0.114	-0.178	0.014		
Triglyceride	0.213	0.040	0.212	0.005		
Total cholesterol	0.011	0.941			0.024	0.830
LDL-C	0.110	0.381	0.114	0.067	0.091	0.401
Met-S score					0.315	<0.001

MDA level was log-transformed (ln) due to nonnormally distribution. Model 1 was the full model with adjusting for waist, LDL-C, TG, HDL-C, total cholesterol, SBP, DBP, and fasting blood sugar. Model 2 was carried out the backward elimination procedure for Model 1. Model 3 was adjusted for Met-S, total cholesterol, and LDL-C. Met-S: metabolic syndrome; HDL-C: high-density lipoprotein cholesterol; LDL-C: low-density lipoprotein cholesterol; MDA: malondialdehyde; SBP: systolic blood pressure; DBP: diastolic blood pressure.

**Table 3:** Associations of uric acid with metabolic components and traditional factors (*n* = 134).

Variables	Model 1		Model 2		Model 3	
	Standardized coefficient beta	*p* value	Standardized coefficient beta	*p* value	Standardized coefficient beta	*p* value
Waist	0.154	0.129				
SBP	-0.063	0.637				
DBP	0.293	0.020	0.275	0.001		
Fasting blood sugar	-0.098	0.285				
HDL-C	-0.174	0.162	-0.204	0.028		
Triglyceride	0.168	0.208	0.171	0.064		
Total cholesterol	-0.040	0.842			-0.047	0.745
LDL-C	0.117	0.470			0.118	0.413
Met-S score					0.415	<0.001

Uric acid level was log-transformed (ln) due to nonnormally distribution. Model 1 was the full model with adjusting for waist, LDL-C, TG, HDL-C, total cholesterol, SBP, DBP, and fasting blood sugar. Model 2 was carried out the backward elimination procedure for Model 1. Model 3 was adjusted for Met-S, total cholesterol, and LDL-C. Met-S: metabolic syndrome; HDL-C: high-density lipoprotein cholesterol; LDL-C: low-density lipoprotein cholesterol; SBP: systolic blood pressure; DBP: diastolic blood pressure.

**Table 4:** Multivariate linear regression analysis of selected risk factors associated with carotid IMT.

Variables	All patients (*N* = 134)		Met − S score = 0 (*N* = 21)		Met − S score ≥ 1 (*N* = 113)		Met − S score ≥ 2 (*N* = 94)		Met − S score ≥ 3 (*N* = 67)		Met − S score ≥ 4 (*N* = 41)	
	Standardized coefficient beta	*p* value	Standardized coefficient beta	*p* value	Standardized coefficient beta	*p* value	Standardized coefficient beta	*p* value	Standardized coefficient beta	*p* value	Standardized coefficients beta	*p* value
Main effect terms												
Uric acid	0.221	0.013	-0.007	0.978	0.292	0.003	0.3295	0.0013	0.3583	0.0016	0.4107	0.0079
MDA	0.115	0.188	0.053	0.830	0.180	0.043	0.1861	0.0478	0.0970	0.3033	0.1239	0.3708
hs-CRP	-0.023	0.771	-0.048	0.843	0.020	0.807	-0.0126	0.8880	-0.0478	0.6080	0.0303	0.8270
Met-S score	0.197	0.044										

Multivariate linear regression model was adjusted for age, gender, smoking, alcohol, uric acid, Met-S score, MDA, and hs-CRP. Met-S: metabolic syndrome; hs-CRP: high-sensitivity C-reactive protein; MDA: malondialdehyde.

## Data Availability

The experimental data and materials used to support the findings of this study are available from the corresponding author upon request.
